# Lack of Adipocyte-Fndc5/Irisin Expression and Secretion Reduces Thermogenesis and Enhances Adipogenesis

**DOI:** 10.1038/s41598-017-16602-z

**Published:** 2017-11-24

**Authors:** D. Pérez-Sotelo, A. Roca-Rivada, I. Baamonde, J. Baltar, A. I. Castro, E. Domínguez, M. Collado, F. F. Casanueva, M. Pardo

**Affiliations:** 1 Grupo Obesidómica, Área de Endocrinología, Instituto de Investigación Sanitaria de Santiago de Compostela (IDIS), Xerencia de Xestión Integrada de Santiago (XXIS/SERGAS), Santiago de Compostela, Spain; 2 Servicio de Cirugía General, Xerencia de Xestión Integrada de Santiago (XXIS/SERGAS), Santiago de Compostela, Spain; 3 CIBER Fisiopatología Obesidad y Nutrición, Instituto de Salud Carlos III, Santiago de Compostela, Spain; 4 Grupo Biofarma C026, Centro de Investigación en Medicina Molecular e Enfermidades Crónicas (IDIS-CIMUS), Universidade de Santiago de Compostela, Santiago de Compostela, Spain; 5 Grupo Células Madre en Cáncer y Envejecimiento, Área de Oncología, Instituto de Investigación Sanitaria de Santiago de Compostela (IDIS), Xerencia de Xestión Integrada de Santiago (XXIS/SERGAS), Santiago de Compostela, Spain; 6 Grupo Endocrinología Molecular y Celular, Instituto de Investigación Sanitaria de Santiago (IDIS), Xerencia de Xestión Integrada de Santiago (XXIS/SERGAS), Santiago de Compostela, Spain

## Abstract

Irisin is a browning-stimulating molecule secreted from the fibronectin type III domain containing 5 precursor (FNDC5) by muscle tissue upon exercise stimulation. Despite its beneficial role, there is an unmet and clamorous need to discern many essential aspects of this protein and its mechanism of action not only as a myokine but also as an adipokine. Here we contribute to address this topic by revealing the nature and role of FNDC5/irisin in adipose tissue. First, we show that FNDC5/irisin expression and secretion are induced by adipocyte differentiation and confirm its over-secretion by human obese visceral (VAT) and subcutaneous (SAT) adipose tissues. Second, we show how secreted factors from human obese VAT and SAT decrease PGC1α, FNDC5 and UCP1 gene expression on differentiating adipocytes; this effect over UCP1 is blunted by blocking irisin in obese secretomes. Finally, by stable gene silencing FNDC5 we reveal that FNDC5-KO adipocytes show reduced UCP1 expression and enhanced adipogenesis.

## Introduction

Recent developments on pursuing the molecular mechanisms implicated in physical activity-induced health benefits, have revealed the discovery of irisin as a muscle-derived factor presumably secreted after the scission of the extracellular portion of the type I membrane protein fibronectin type III domain containing protein 5 (Fndc5)^1^. The significance of this finding is built on the beneficial effects recognized to this myokine. Hence, it was described that upon exercise stimulation, and through the transcriptional co-activator PGC1α, the expression of FNDC5 is increased in muscle and irisin secreted, inducing the stimulation of thermogenesis genes in certain adipocytes^1,2^. Accordingly, irisin may behave as a muscle-derived energy-expenditure signal that directly communicates with adipose tissue inducing browning. This role attributed to irisin would be responsible for a white adipose tissue (WAT) metabolic profile improvement, raising whole-body energy expenditure. Moreover, a number of evidences suggest that irisin improves glucose homeostasis, and that it’s circulating levels show an inverse association with liver fat content^3–5^. Thus, irisin was revealed as a potential new target for the treatment of metabolic diseases.

In contrast with the above, other investigations are questioning the primary beneficial role of irisin or even its existence generating a great debate in the literature^6–8^. First, there is disagreement regarding the regulation of FNDC5/irisin by exercise^9,10^; and surprisingly, it was described that circulating irisin levels in humans are positively correlated with parameters of adiposity, finding the highest levels in obese individuals^9,11,12^. Further evidences showed an association of irisin circulating levels with markers of glucose and lipid homeostasis disturbance in obesity and with metabolic syndrome^13–16^.

We latterly discovered that FNDC5/irisin is also an adipokine expressed and secreted by visceral and especially by subcutaneous adipose tissue in rats under *ad libitum* conditions^17^. In this study, we observed that the secretion of FNDC5/irisin by both adipose depots was increased by short-term exercise and reduced by fasting. Interestingly, and contrary to expected, we observed that obese adipose tissue over-secreted this protein compared to equivalent tissue from lean individuals. Paralleling the pre-clinical results, we further described that adipose tissue of human origin and to a greater extent from obese subjects, could also express and contribute to circulating FNDC5/irisin^11,12^. Besides, we found an association between the reduction of plasma irisin levels and the depletion of lipid metabolism biomarkers in patients with metabolic syndrome under an energy-restricted dietary program^14^, and a possible role of circulating irisin as a predictor of the insulin resistance onset in association with weight regain in obese individuals^13^. On the whole, the mentioned data supports the hypothesis that increased circulating irisin, probably secreted by adipose tissue, may be an adaptive response to counterbalance decreased insulin sensitivity and other metabolism disorders associated with obesity^3,9^. We suggest a physiological feedback that is increased in unfavorable metabolic situations generating a compensatory mechanism that may recede once the altered metabolic state is restored as it occurs after weight loss^11^.

In the present paper we go a step forward by revealing the increasing expression and secretion of FNDC5/irisin during adipocyte differentiation, elucidating FNDC5/irisin secreted isoforms in adipocytes, demonstrating the elevated secretion of this adipokine by human obese adipose VAT and SAT explants, revealing the negative effect of obese adipose secreted factors on FNDC5 expression on normal adipocytes, and more importantly, achieving a FNDC5-KO murine adipocyte cell line that shows decreased UCP1 expression and enhanced adipogenesis.

## Results

### Differentiated C3H10T1/2 cells express and secrete FNDC5/irisin

**Figure 1:**
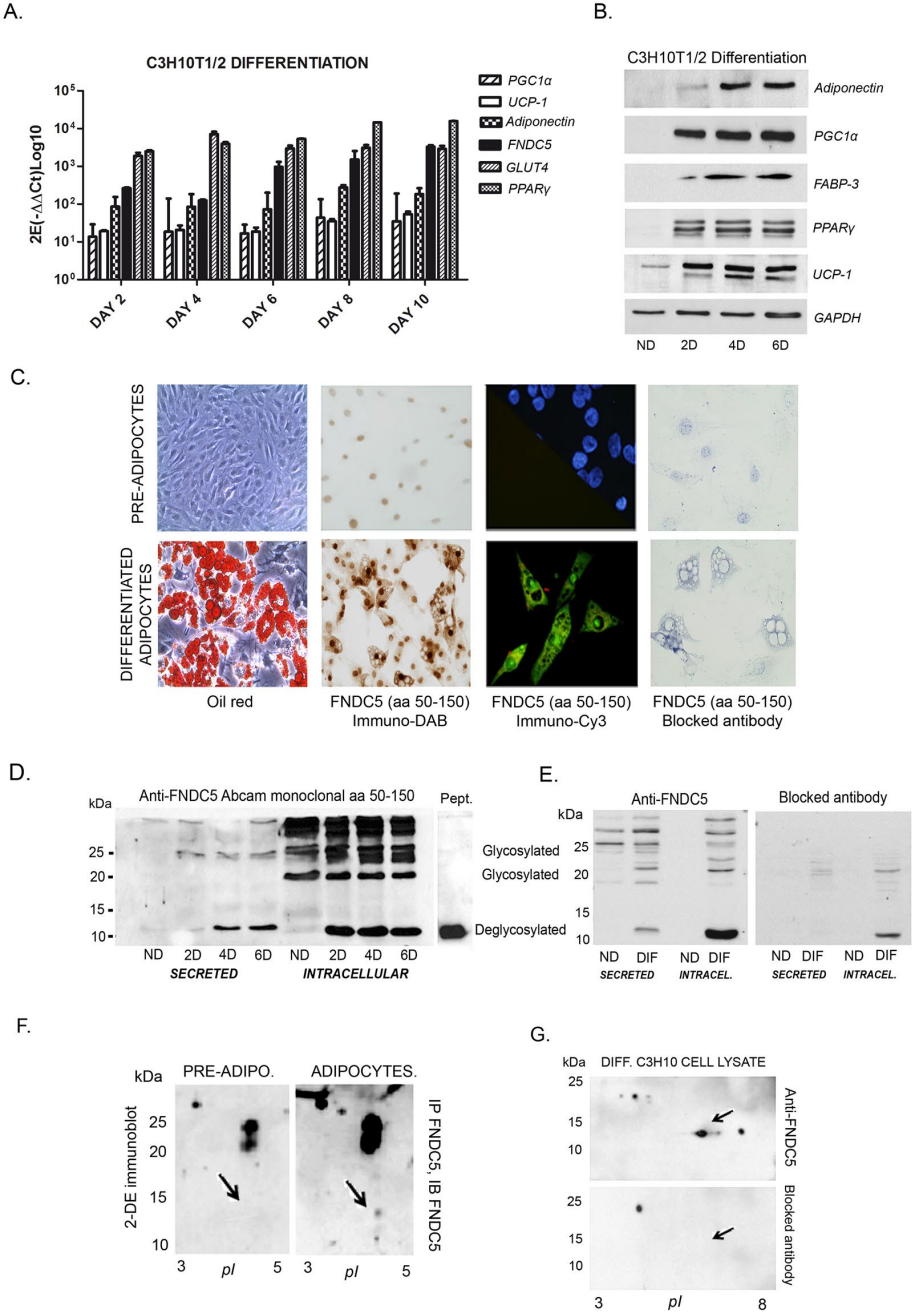
Only differentiated C3H10T1/2 murine mesenchimal stem cells express and secrete FNDC5/irisin. (**A**,**B**) mRNA expression and representative protein immunoblots of differentiation markers throughout differentiation of C3H10T1/2 cells. 2E(-∆∆Ct) and error values and representative immunoblot film images from where figures where cropped are shown in Supplementary Figure 5; (**C**) representative images of pre-adipocytes and mature C3H10T1/2 cells stained with oil red or analyzed for FNDC5 detection by immunocitochemistry (DAB) and immunofluorescence (Cy3) with and without blocked antibody; (**D**) representative immunoblot against FNDC5/irisin in differentiated and non-differentiated cells showing all the detected bands along differentiation at secreted and intracellular level; (**E**) FNDC5 immunoblot of secretomes and cellular lysates from pre-adipocytes and mature differentiated cells at day 6 with and without blocked FNDC5 antibody; (**F**) 2-DE immunoblot of immunoprecipitated FNDC5 from pre-adipocytes and mature differentiated cells secretomes showing a 12 kDa spot only present in adipocytes (pointed with and arrow). Note that representative immunoblot images have been cropped from the film in Supplementary Figure 6A; (**G**) image showing irisin spot disappearance pointed with and arrow after using a blocked antibody. Note that representative immunoblot images have been cropped from the film in Supplementary Figure 6B. DAB, diaminobenzidine; Pept: commercial irisin peptide.

To characterize FNDC5/irisin on adipose tissue, a first analysis was performed in C3H10T1/2 cells by studying the expression of FNDC5 from a non-differentiated state throughout adipogenesis. FNDC5 gene and protein expression was only detected in differentiated cells with increasing expression from day 2 to 10 paralleling the expression of PPARγ, GLUT4, UCP1, adiponectin and PGC1α (Fig. 1A,B). Further, FNDC5 protein detection by immunohistochemistry was observed exclusively in differentiated C3H10T1/2 cells (Fig. 1C).

Immunoblotting analysis of C3H10T1/2 intracellular and secreted fractions using a monoclonal antibody against FNDC5 (aa 50–150) showed different bands at diverse molecular weights (Fig. 1D). Thus, antibody incubation with a specific blocking peptide showed unspecific binding at the high molecular weight area (Fig. 1E). Interestingly, a band compatible with the described secreted portion of FNDC5, known as irisin, was detected at 12 KDa in secretomes of differentiated cells (Fig. 1D). The presence of this form follows the gene expression profile, being absent in pre-adipocytes secretomes with increasing presence from differentiation day 2 (Fig. 1D). A faint band at 25 kDa was also detected in the cell culture medium of differentiated cells. At intracellular level, bands at 25, 20 and 12 kDa were assumed as specific paralleling previously described irisin glycosylated isoforms, irisin dimmers and/or complete FNDC5 protein (Fig. 1D). FNDC5 immunoprecipitation in C3H10T1/2 cells secretomes followed by two-dimensional (2-DE) western blotting confirmed the previous immunoblots by detecting an exclusive spot in differentiated adipocytes with the expected molecular weight and isoelectric point for 12 kDa-secreted non-glycosylated irisin (Fig. 1F, marked with an arrow). 2-DE western blot showed specific spots at 12 kDa that disappear using blocked antibody also in cell lysates (Fig. 1G, marked with an arrow).

### Human visceral and subcutaneous adipose tissues express and secrete FNDC5/irisin especially in obesity

**Figure 2:**
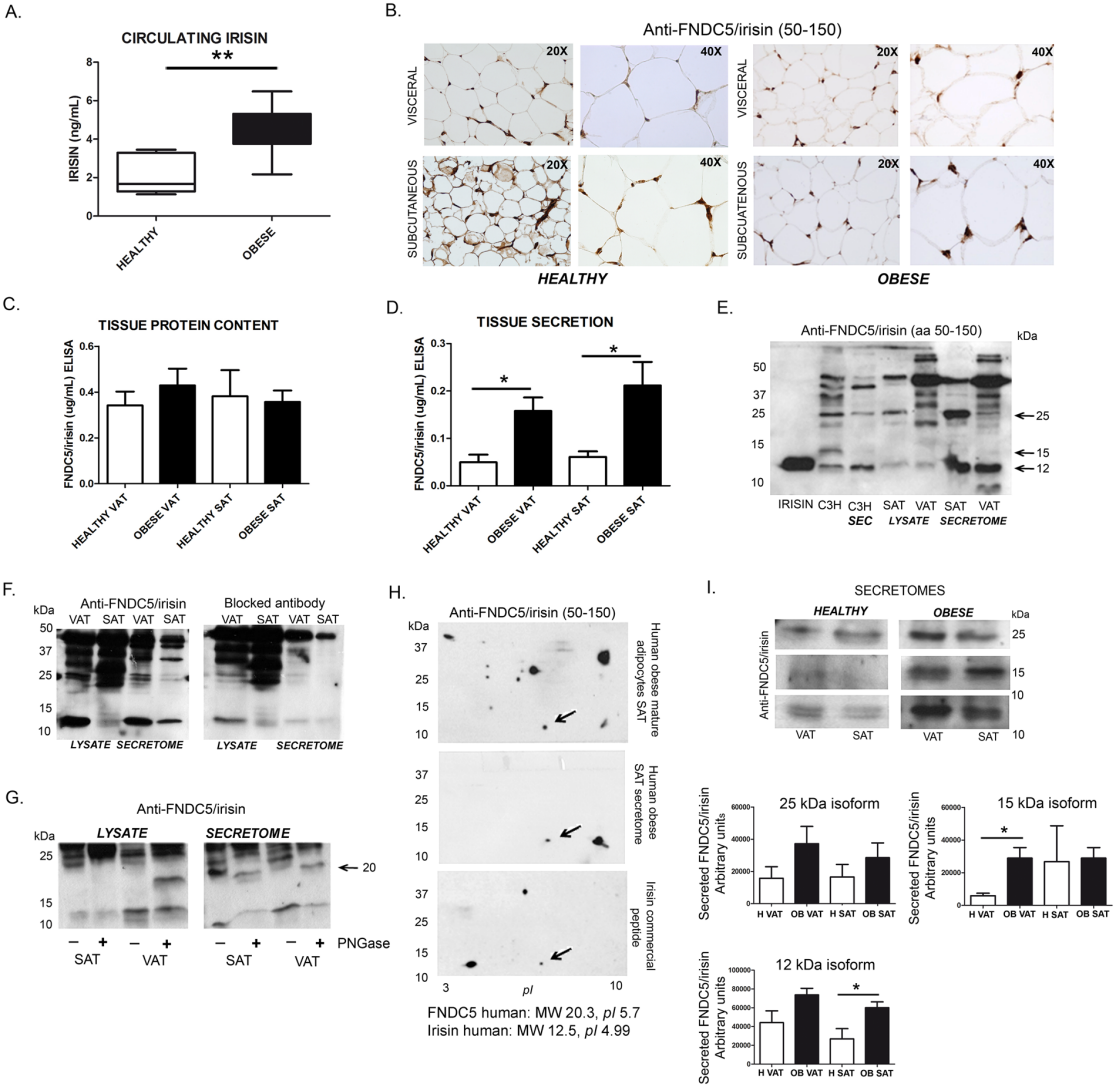
FNDC5/irisin is expressed and secreted by human adipose tissue. (**A**) Comparative irisin circulating levels between healthy and obese individuals selected for FNDC5/irisin tissue analysis [healthy (*n* = 6) vs. obese (*n* = 17) p = 0.0007] Mann-Whitney *U* test; (**B**) Detection of FNDC5/irisin in representative images of human obese and healthy VAT and SAT sections. (**C**) FNDC5/irisin protein content quantification by ELISA in human obese (*n* = 17) and healthy (*n* = 4) VAT and SAT lysates [p = ns; Mann-Whitney *U* test]; (**D**) FNDC5/irisin *in vitro* direct protein secretion quantified by ELISA from human obese (*n* = 35) and healthy (*n* = 4) VAT and SAT explants cultured *in vitro* [HVAT vs OBVAT p = 0.028; HSAT vs OBSAT p = 0.0045]; Mann-Whitney *U* test; (**E**) Representative immunoblot showing FNDC5/irisin band pattern of murine cells and human samples lysates and secretomes; (**F**) Representative image of FNDC5/irisin detection in human VAT and SAT lysates and the signal using a blocked antibody; (**G**) FNDC5/irisin band shift detection of human SAT and VAT lysates and secretomes treated with PNGase; (**H**) 2-DE FNDC5 immunoblots of human obese mature SAT adipocytes lysate, their secretome, and 2-DE detection pattern of irisin commercial peptide; (**I**) Representative immunoblots of the most prominent FNDC5/irisin bands detected in human VAT and SAT explants secretomes. Note that representative immunoblot images have been cropped from the film in Supplementary Figure 7. Band quantification of at least 4 independent patients is represented in histograms [15 kDa HVAT vs OBVAT p = 0.0364; 12 kDa HSAT vs OBSAT p = 0.0341] Mann-Whitney *U* test. *pI*: isoelectric point.

Human visceral (VAT) and subcutaneous (SAT) adipose tissues from healthy and obese individuals were analyzed for FNDC5/irisin protein expression and secretion. Firstly, circulating irisin levels of these patients were tested finding, as we previously described in different cohorts of patients^11–13^, elevated circulating irisin levels in obese patients compared to their lean counterparts (Fig. 2A). Although tissue protein content studies by immunohistochemistry showed a positive staining in both VAT and SAT depots, there were no apparent differences between healthy and obese adipose tissues (Fig. 2B). Further, irisin quantification in human VAT and SAT at intracellular level by ELISA confirmed no statistical differences (Fig. 2C). Interestingly, a clear and significant over-secretion was observed in VAT and SAT secretomes from obese subjects compared to those from lean individuals (Fig. 2D).

Considering the uncertain specificity of commercially available ELISA kits against irisin, immunoblot analysis were additionally performed. Thus, FNDC5/irisin detection in VAT and SAT lysates and secretomes from obese individuals confirmed the presence of FNDC5/irisin in human adipose samples and a similar band pattern to that of C3H10T1/2 cells (mouse and human irisin are 96.698% identical-Supplementary Figure 1). Loading C3H10T1/2 murine samples and human VAT and SAT together in the same blot, showed no differences on the 12 kDa-irisin band that migrated at the same molecular weight as irisin commercial peptide (Fig. 2E). Thus, unspecific antibody binding was detected in the high molecular weight area, and a prominent band at 12 kDa, that disappears or diminish its intensity by blocking the detection antibody, was observed (Fig. 2F). This 12 kDa band was not detected in the stromal vascular fraction (SVF) of human VAT and SAT (Supplementary Figure 2). Other bands at 15 and 25 kDa were also detected in VAT and SAT lysates and secretomes (Fig. 2F). Interestingly, the 25 kDa band observed in both VAT and SAT lysates and secretomes shifted to approximately 20 kDa after PNGase treatment confirming N-linked glycosylation (Fig. 2G). Isolation of SAT mature adipocytes and their secretome allowed us to characterize human FNDC5/irisin isoforms by two-dimensional western blot using an irisin commercial peptide as a reference (Fig. 2H). In the former analysis, a spot at 12 kDa and an isoelectric point of 5 was found common to SAT lysate, secretome and commercial peptide samples (marked with an arrow in Fig. 2H).

Further band quantification of the secreted FNDC5/irisin specific isoforms at 25, 15 and 12 kDa showed a prevalent elevation of the three isoforms in VAT and SAT secretomes from obese patients that become significant in 15 kDa obese VAT and in 12 kDa obese SAT compared to their respective healthy adipose tissue (Fig. 2I).

### Obese VAT and SAT secreted factors reduce FNDC5 and UCP1 gene expression of healthy adipocytes

**Figure 3:**
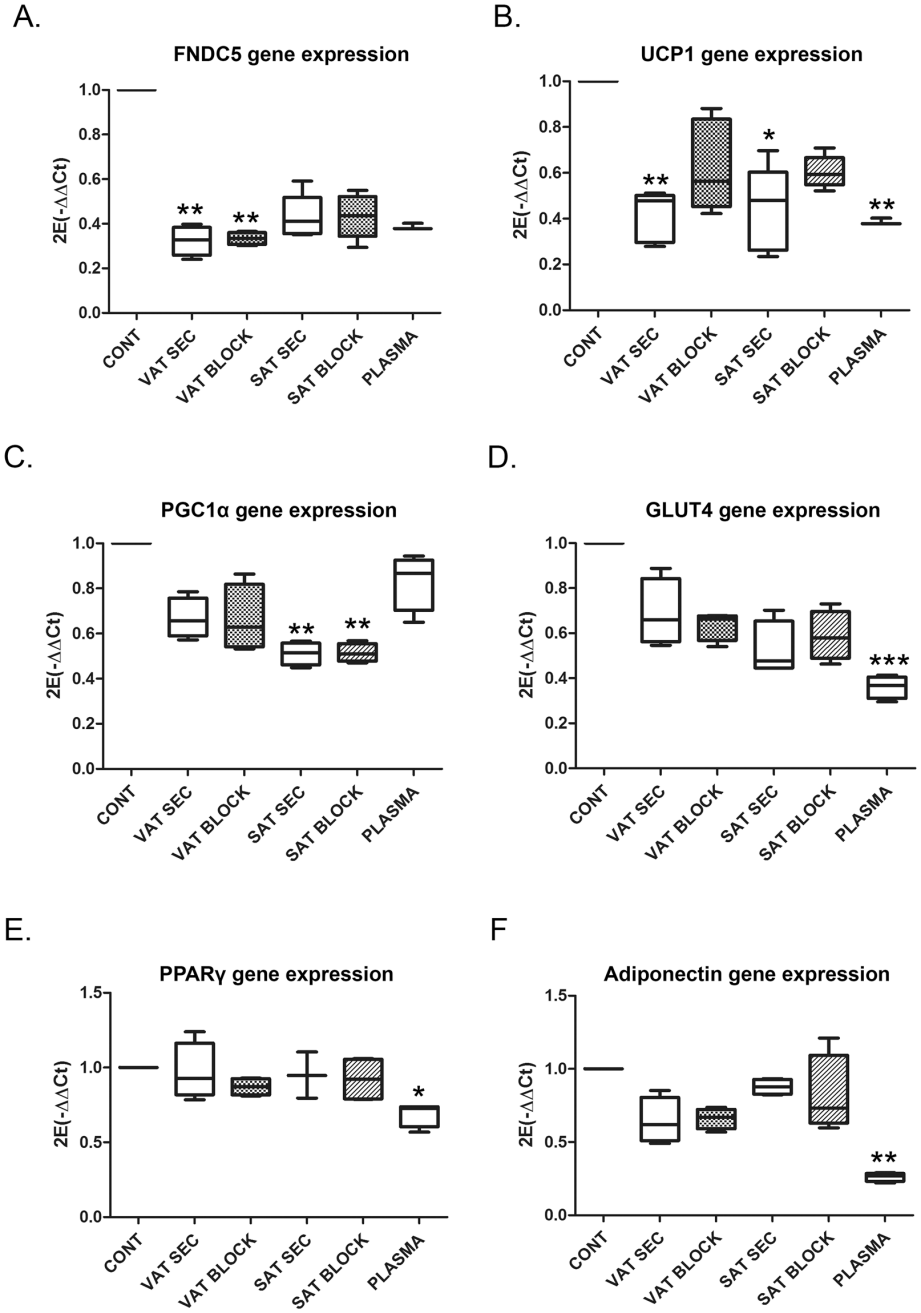
Human obese VAT and SAT secreted factors alters normal adipose cells gene expression during differentiation. (**A**) FNDC5; (**B**) UCP1; (**C**) PGC1α; (**D**) GLUT4; (**E**) PPARγ; and (**F**) Adiponectin gene expression in murine C3H10T1/2 pre-adipocytes (day 2) differentiated in the presence of obese VAT and SAT secreted factors with or without blocking soluble FNDC5/irisin during 24 hours (*n* = 4 independent human obese secretomes). The effect of plasma from the same obese patients is also shown for each analyzed gene. Histograms show the quantitative expression levels towards control non-treated cells for data normalization [One way ANOVA Kruskal-Wallis test followed by Dunn’s multiple comparison]. *p < 0.05, **P < 0.01, and ***p < 0.001 versus control non-treated cells. CONT: cells without treatment; VAT and SAT SEC: cells treated with 10% obese SAT or VAT secretome; VAT and SAT BLOCK: cells treated with obese VAT and SAT secretome with antibody blocked-irisin; PLASMA: cells treated with 10% obese plasma.

To test the effect of obese adipose tissue secreted factors on FNDC5 gene expression, C3H10T1/2 cells were committed to differentiation and incubated from day 2 in the presence of obese VAT and SAT conditioned medium with or without blocking FNDC5/irisin protein with a blocking antibody. The incubation with obese SAT and VAT secretomes reduced FNDC5 gene expression (Fig. 3A); however, only VAT and irisin-depleted VAT secretome conditioned medium achieved a significant reduction (**p < 0.01). Further gene expression analysis showed a significant inhibition of UCP1 gene expression by both obese VAT (**p < 0.01) and SAT (*p < 0.05) secretomes. This UCP1 gene expression inhibition by obese VAT and SAT secretomes was reversed by blocking irisin in both cases (Fig. 3B).

PGC1α gene expression was also tested in the same experimental setting showing a significant reduction in the presence of obese SAT secretome independently of irisin (**p < 0.01) (Fig. 3C). No effect was observed in GLUT4, PPARγ and adiponectin gene expression (Fig. 3D,F). Adipocyte differentiation in the presence of plasma from the same obese patients, showed a significant reduction of UCP1 (**p < 0.01), GLUT4 (***p < 0.001), PPARγ (*p < 0.05) and adiponectin (**p < 0.01) gene expression compared to control cells (Fig. 3A–F).

The same experiment performed with VAT and SAT secretomes from healthy lean donors showed no significant effect on the same genes expression (Supplementary Figure 3).

**Figure 4:**
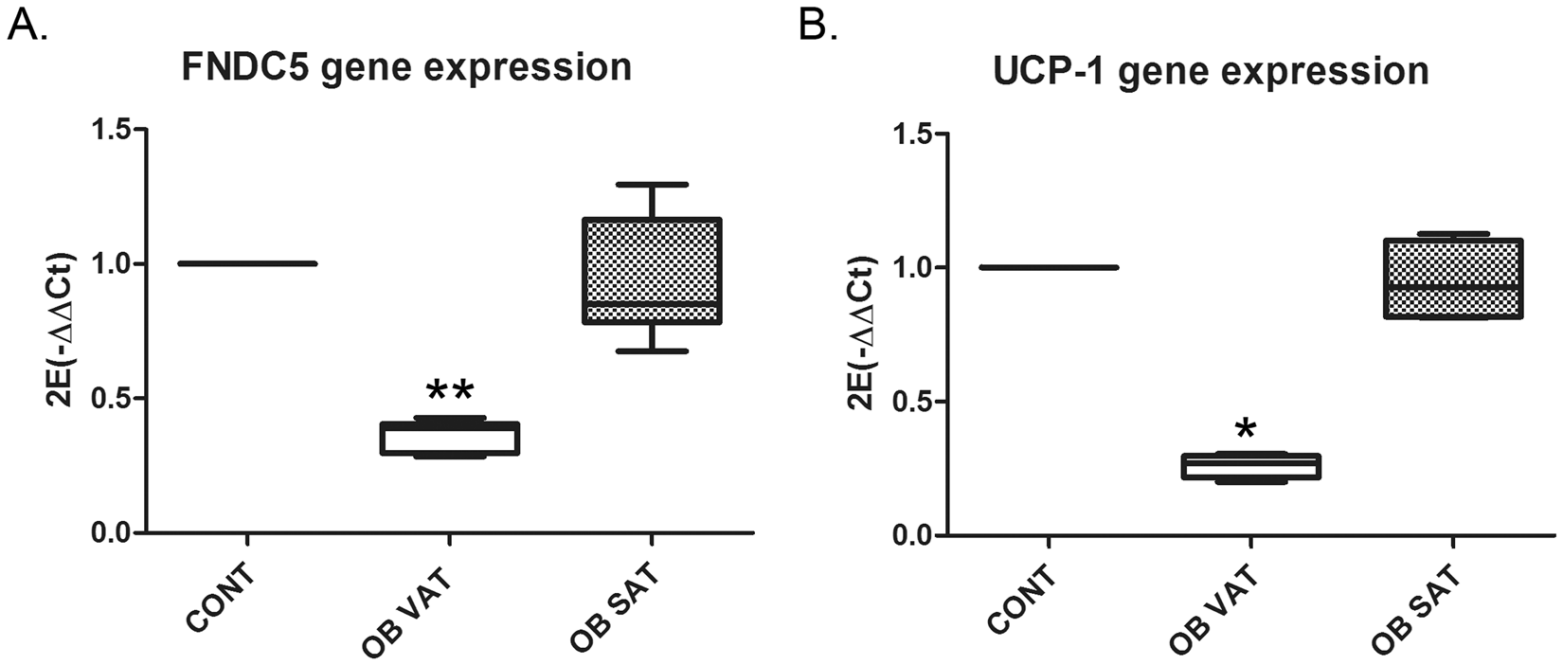
Human obese VAT and SAT secreted factors alters differentiated adipose cells gene expression. (**A**) FNDC5, and (**B**) UCP1 gene expression in murine C3H10T1/2 differentiated adipocytes at day 10 after treatment with obese VAT and SAT secreted factors during 24 hours (n = 4 independent human obese secretomes). Histograms show the quantitative expression levels towards control non-treated cells for data normalization [One way ANOVA Kruskal-Wallis test followed by Dunn’s multiple comparison]. *p < 0.05 and **P < 0.01 versus control non-treated cells. CONT: differentiated cells without treatment; OB VAT/SAT: differentiated cells treated during 24 hours with obese VAT and SAT secretomes.

Finally, to test the effect of obese secreted factors on FNDC5 once adipocytes are totally differentiated, the expression of FNDC5 together with UCP-1 was assayed by exposing adipocytes at day 10 of differentiation to obese VAT and SAT secretomes during 24 hours. Under this situation, only obese VAT secreted factors could reduce significantly FNDC5 (**p < 0.01) and UCP-1 gene expression (* p < 0.05) (Fig. 4).

### Adipose FNDC5 gene silencing show specific irisin signal

**Figure 5:**
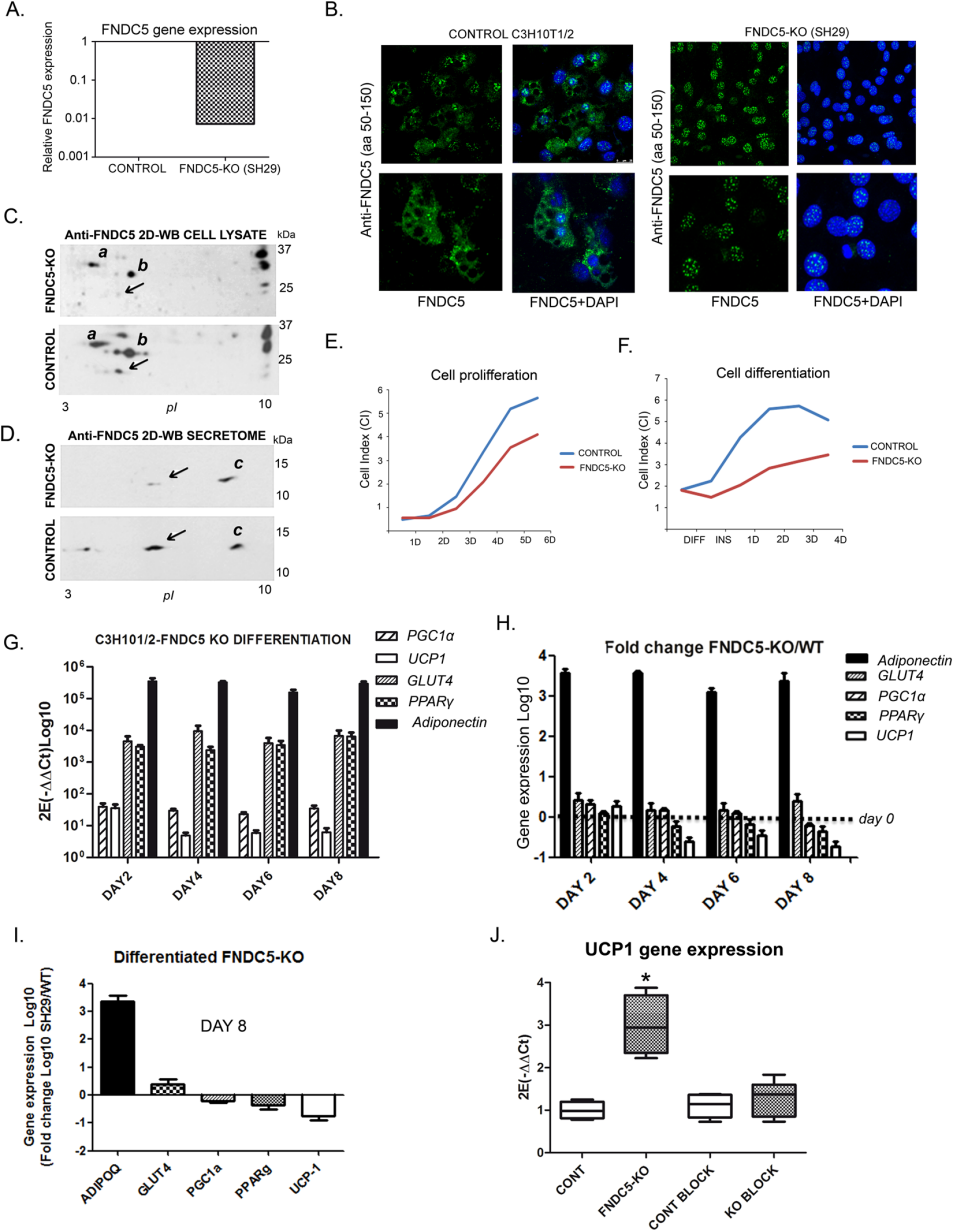
FNDC5 silencing in C3H10T1/2 adipocytes. (**A**) FNDC5 gene expression inhibition compared to control cells; (**B**) Representative images of FNDC5 immunofluorescent staining in control and FDNC5 silenced cells (FNDC5 in green; nuclear staining by DAPI in blue); (**C**) 2-DE immunodetection of FNDC5/irisin in control and FNDC5-KO cell lysates. Note that representative immunoblot images have been cropped from the film in Supplementary Figure 8A and B; (**D**) 2-DE immunodetection of FNDC5/irisin in control and FNDC5-KO cells secretomes. Note that representative immunoblot images have been cropped from the film in Supplementary Figure 8C and D; (**E**) Real time proliferation measurement of control vs. FNDC5-KO cells during 6 days; (**F**) Real time differentiation monitoring of control vs. FNDC5-KO cells from non differentiated state to differentiation day 4; (**G**) mRNA expression of differentiation markers throughout differentiation of FNDC5-KO C3H10T1/2 cells; (**H**) Differentiation markers expression fold change of FNDC5-KO cells over wild type; (**I**) Gene expression fold change at day 8; (**J**) UCP1 gene expression of control and FNDC5-KO cells differentiated in the presence of intact wild type secretome or blocked with anti-FNDC5 antibody (*n* = 4 independent experiments; p = 0.174) One way ANOVA Kruskal-Wallis test followed by Dunn’s multiple comparison.

To reveal the functional role of FNDC5/irisin in adipose tissue, C3H10T1/2 cells were knocked down for FNDC5 expression by shRNA with lentiviral infection. From five different shRNAs, FNDC5 sh29 was selected as with the highest gene expression inhibition without affecting the intrinsic capacity to be differentiated into mature adipocytes (Fig. 5A). Immunodetection of FNDC5 in silenced cells after complete differentiation showed a strong nuclear positive staining with no cytoplasmic or cell membrane signal compared to control cells that showed both nuclear and cytoplasmic staining (Fig. 5B). 2-DE immunoblot analysis of silenced cell lysates shows that the monoclonal FNDC5 antibody binds to proteins other than FNDC5/irisin (spots *a* and *b* Fig. 5C). The spot with the FNDC5 expected isoelectric point and molecular weight tagged with an arrow (mouse FNDC5 predicted at *Uniprot*: 20.3 kDa and 5.7 *pI*) disappears after FNDC5 gene silencing (Fig. 5C). Moreover, the 2-DE immunoblot of FNDC5-KO cells secretome shows that the signal of the spot tagged with an arrow, that corresponds with the expected secreted irisin peptide (mouse irisin predicted at *Uniprot*: 12.6 kDa and 4.99 *pI*), diminishes significantly its signal from the blot, while no variation was shown in a more basic spot also detected with the irisin antibody (Fig. 5D, labeled as c). Accordingly, this same spot is present in the 2-DE immunoblot of commercial irisin shown in Fig. 2H.

### Adipocytes lacking FNDC5 gene expression show reduced UCP1 expression and increased adipogenesis

**Figure 6:**
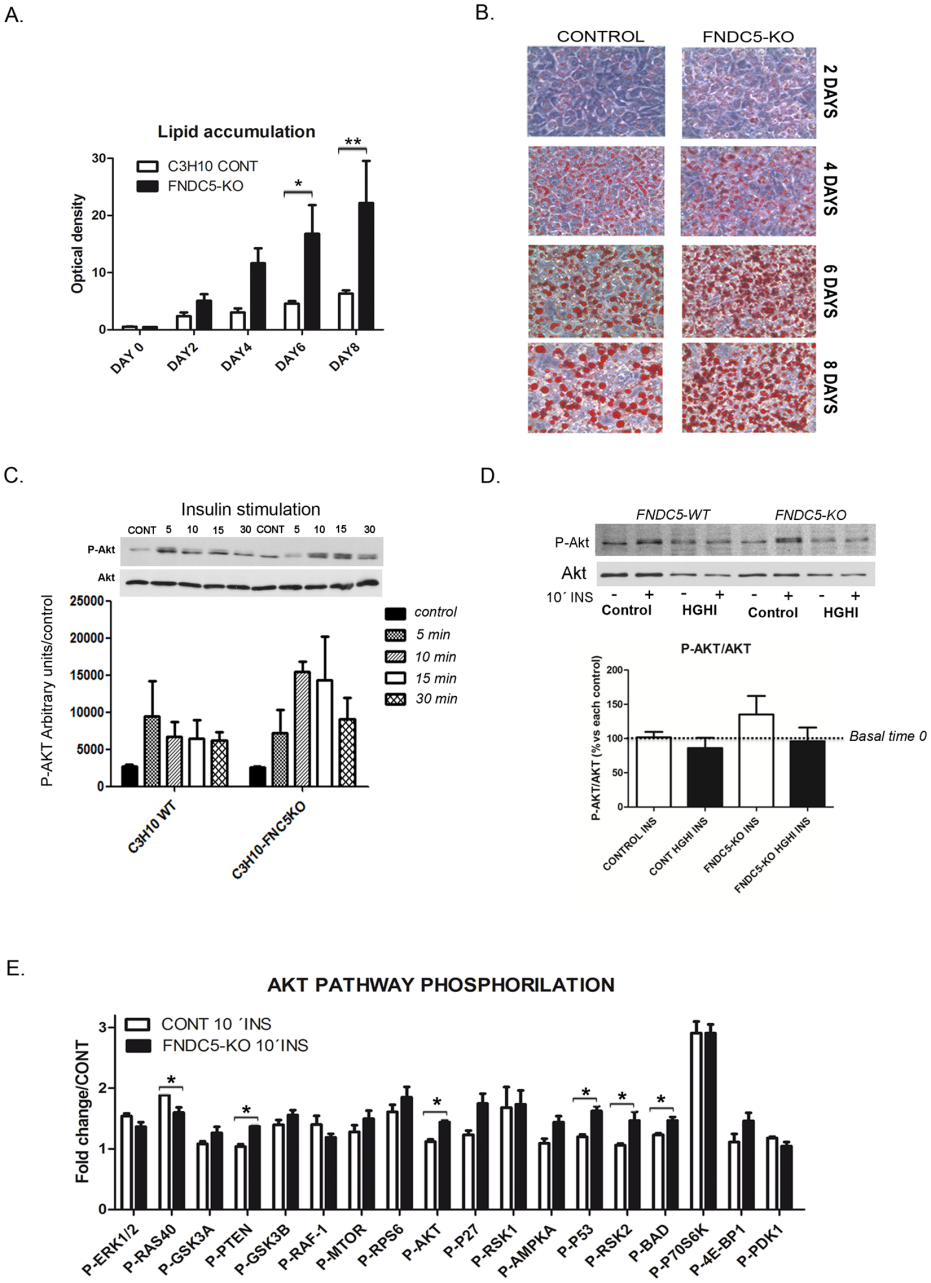
Functional analysis of FNDC5-KO adipocytes. (**A**) Oil red quantification of lipid accumulation along differentiation of control vs. FNDC5-KO adipocytes (*n* = 3 independent differentiation experiments) Two-way ANOVA followed by Bonferroni post hoc; (**B**) Representative images of control and FNDC5-KO cells stained with oil red until differentiation day 8; (**C**) Insulin sensitivity of control and FNDC5-KO cells assayed by P-Akt levels immunodetection after insulin time course stimulation; (**D**) Insulin resistance assay after high glucose/high insulin treatment. Representative immunoblots of control and FNDC5-KO cells stimulated during 10 minutes with insulin. Data is represented towards basal P-Akt levels (time 0) and corrected towards total Akt (n = 3 independent experiments) One way ANOVA Kruskal-Wallis test followed by Dunn’s multiple comparison. (**E**) Protein phosphorylation array analysis showing the activation of 18 proteins under Akt pathway, after 10 min insulin (100 mM) stimulation (*n* = 4 independent experiments) Mann-Whitney *U* test [p-RAS40 p = 0.0265; p-PTEN p = 0.0294; p-Akt p = 0.286; p-P53 p = 0.0286; p-RSK2C p = 0.0286; p-BAD p = 0.286]. Statistical significance is represented as *p < 0.05, **P < 0.01, and ***p < 0.001.

Further analysis of FNDC5-KO (C3H10sh29) cells by real time monitoring showed a decreased cell index compared to control cells during proliferation and differentiation (Fig. 5E,F). Thus, a two days advantage on adipocyte differentiation was observed in FNDC5-KO cells. Gene expression analysis of these cells showed a gradual UCP1 gene expression reduction throughout differentiation, and a high adiponectin expression from day 2 (Fig. 5G). A comparative gene expression analysis during differentiation showed that FNDC5-KO cells have a gradual decreased expression level of UCP1 compared to control cells (Fig. 5H). On the other hand, cells lacking FNDC5 showed elevated levels of adiponectin and a slight elevation of GLUT4 compared to wild type adipocytes at differentiation day 8 (Fig. 5I). Interestingly, differentiation of FNDC5-KO cells in the presence of irisin-containing control cells secretome restored UCP1 diminution by significantly increasing its expression (*p < 0.05); this effect was reverted by blocking irisin present in the secretome with a monoclonal antibody (Fig. 5J). Relevantly, we observed that FNDC5-KO cells accumulate noticeable more lipids than control cells during differentiation becoming significant from day 6 (Fig. 6A,B).

### FNDC5-KO adipocytes show changes in phospho-Akt pathway

To get a deeper insight into the functional role of FNDC5/irisin in adipose tissue, further analyses were performed to assess the glucose metabolism of FNDC5-KO cells. Thus, insulin sensitivity was tested finding a 5 minutes delay to reach the highest signal assayed by p-Akt detection compared to control cells (Fig. 6C). Induction of insulin resistance by treating cells with a combination of high glucose and high insulin (HGHI) showed that cells lacking FNDC5 are not more resistant to insulin than control cells after 10 minutes insulin stimulation (Fig. 6D).

The assessment of phospho-Akt pathway activation after 10 minutes insulin treatment confirms a significant increase of Akt (Ser473) in FNDC5-KO cells compared to control cells. Further, a decrease in RAS40 (P-Thr246) and an augmentation of PTEN (P-Ser380), P53 (P-ser15), RSK2 (P-Ser386) and BAD (Ser112) was found (Fig. 6E).

### FNDC5-KO adipocytes are not sensitive to obese adipose secretome

Contrary to normal cells in Fig. 3, FNDC5-KO adipocytes showed no changes on UCP1 expression in the presence of obese VAT and SAT human secretomes, neither with healthy lean secretome samples (Supplementary Figure 4A,B). Additionally, no changes were found in PPARγ and adiponectin gene expression (Supplementary Figure 4E–H). Only PGC1α and GLUT4 gene expression changes were observed. PGC1α expression was significantly reduced by differentiating the cells in the presence of healthy VAT secretome, and by treating them with healthy SAT containing blocked-irisin (HSAT BLOCK) (Supplementary Figure 4C); moreover, a significant reduction of GLUT4 expression was detected after treating FNDC5-KO cells with lean and obese VAT and SAT secretomes independently of irisin (Supplementary Figure 4I,J).

## Discussion

In this work, we significantly contribute to many of the unsolved concerns about FNDC5/irisin by successfully revealing the nature and role of this protein in adipose tissue. We continue with our previous discovery describing FNDC5/irisin as an adipokine over-secreted by obese adipose tissue, which lead us to hypothesise that adipose-derived FNDC5/irisin might participate on the elevated irisin levels detected on obese individuals^5,10–14,16^. Firstly, we describe for the first time the progressively expression and secretion of FNDC5/irisin throughout pre-adipocyte differentiation getting to the highest levels upon reaching the mature differentiated state. By direct irisin quantification on secretomes from human adipose tissue explants we confirm that obese visceral (VAT) and subcutaneous (SAT) adipose tissues secrete more irisin than healthy adipose tissues. The immunoblot analysis of adipose tissue lysates and secretomes, using to our experience the best antibody available, shows three predominant bands at 25, 15 and 12 kDa, being the 12 kDa band the most abundant in secretomes. All three isoforms were found elevated in secretomes from human obese VAT and SAT without significant differences among tissue depots. Secondly, we show how autocrine secreted factors from human obese VAT and SAT depots decrease PGC1α, FNDC5 and UCP1 gene expression on murine differentiating adipocytes; this negative effect over UCP1 expression is blunted by blocking irisin in obese VAT and SAT secretomes. Finally, we are the first to achieve the stable silencing of FNDC5 gene on cultured pre-adipocytes. This ultimate approach, besides allowing the identification of the authentic FNDC5/irisin protein signal that confirms the unspecific binding of commercial antibodies, reveals an important role for FNDC5 in adipogenesis. Hence, cells lacking FNDC5 expression show reduced UCP1 expression and increased adipogenesis confirmed by significantly higher lipid accumulation during differentiation. Interestingly, cells lacking FNDC5/irisin although show a delayed response to insulin stimulus compared to control cells, they do not evidence a clear insulin resistance.

Despite the great excitement originated by the discovery of FNDC5/irisin as a browning-stimulating myokine secreted by muscle tissue upon exercise stimulation; there are still many unknown and essential aspects of this protein and its mechanism of action not only as a muscle secreted factor but also as an adipokine. At this moment, with an exponential number of published papers in this topic, there is an urgent need to discern the regulation and nature of irisin secretion, the functional role of its post-translational modifications, its secretion profile and actions under healthy and pathological situations, and the discovery of the pathways stimulated upon binding to its still unknown receptor. Under this context, there is a crucial topic concerning the specificity of the anti-FNDC5/irisin commercially available antibodies that has generated uncertainty in regard to many of the published results^5,7^.

In the present manuscript, we demonstrate the expression and secretion of FNDC5/irisin by adipose cells and tissues revealing an expression and secretion profile that parallels other adipogenic factors. Likewise, the lack of expression of FNDC5 in non differentiated adipocytes suggests a role of FNDC5/irisin on adipogenesis. It is important to highlight the detection of a 12 kDa irisin band and spot, with the expected isoelectric point and molecular weight, in both murine and human adipose samples. To our knowledge, this is the first time that this band/spot representing the non-glycosylated/non-dimerized form of irisin is shown in human samples by western blotting. Its predominant signal in cell and tissues secretomes suggests a functional role for the non-glycosilated irisin accordingly to the experimental work published elsewhere showing that recombinant 12 kDa irisin exerts biological activity^18–20^. Thus, our data confirms the results by Jedrychowski and collaborators showing the identification by mass spectrometry of a 12 kDa peptide sequenced as irisin in human plasma samples^21^. The former and our study confirm the expression and secretion of FNDC5/irisin in humans; proving that although irisin is mainly translated in humans by a non-canonical start codon^22^, it still reaches significant levels of expression and secretion. We believe that the presence of other bands at 15 and 25 kDa may be explained by modifications such as glycosilation or even dimerization^23^. Under this context, we are the first to accurately demonstrate the unspecific binding of anti-FNDC5/irisin commercial antibodies by creating a FNDC5-KO cell line. Two spots at 20 and 12 kDa, with the FNDC5/irisin predicted isoelectric point and molecular weight, disappear after silencing FNDC5 gene in C3H10T1/2 cells; we suggest that the spots at the same or similar molecular weight recognized by the anti-FNDC5 antibody after silencing might be the explained by the unspecific binding to FNDC4, and probably to its excised soluble form^24^. The use of basic local alignment search tool finds a 53.5% local similarity between FNDC5 and FNDC4 in both human and mouse (Uniprot blast). Accordingly, immunocitochemistry analysis showed a typical nuclear signal in non-differentiated control cells and in FNDC5-KO adipocytes; only differentiated control adipocytes showed cytoplasmic and cell membrane staining which may correspond with the expected FNDC5/irisin. This statement reveals that studies performed with commercial FNDC5 antibodies including ELISA assays should be taken cautiously as previously reiterated^5,7,8^. Standard western blot allows discerning different isoforms but we definitively reveal that at this moment the only way to detect the real FNDC5/irisin is by 2-DE western blotting or directly by mass spectrometry as formerly described^21^.

Accordingly to our results, it is reasonable to suggest that adipose tissue may attempt to tackle obese deregulated metabolism by over-secreting beneficial irisin. The clue in this pathological state resides in the tissue sensitivity to irisin or in the identification of other factors that may negatively regulate irisin effects. It is interesting to emphasize in this respect that our data show that secreted factors from obese adipose tissues decrease PGC1α expression and inhibit FNDC5 gene expression of healthy adipose differentiating cells. Because blocking obese-irisin on the same obese secretomes do not revert FNDC5 and PGC1α gene expression inhibition, we suggest other obese secreted factors as responsible of this effect. Paralleling the inhibition of FNDC5 by obese adipose tissue secreted factors, a significant diminution of UCP1 browning marker was observed in healthy differentiating cells in the presence of obese VAT and SAT. Interestingly, in this occasion, this inhibition was restored by blocking obese-irisin in the obese secretomes. In consequence, UCP1 gene expression was found to be downregulated compared to control cells in FNDC5 silenced adipocytes. Further incubation of FNDC5-KO cells with control cell secretomes, containing healthy irisin, restored UCP1 gene expression in these cells. This at first sight contradictory effect of irisin on UCP1 expression can be explained by a recent report showing that irisin inhibits UCP1 gene expression during adipocyte differentiation, and only after formation of mature adipocytes is able to exert this browning effect^25^. All together, these findings are coherent with a positive regulation of thermogenesis initially attributed to irisin^1,26^ which may be challenged in the occurrence of obesity probably due to other interfering factors^27^. Thus, we observed that circulating factors present in the plasma of obese individuals reduce the expression of FNDC5, UCP1, GLUT4, PPARγ and adiponectin in cultured healthy murine adipocytes.

It is of special interest the functional studies of irisin on adipose cells by stable silencing FNDC5 expression. To our knowledge, this is the first time that FNDC5 expression is silenced in adipocytes. This experimental approach responds to experts in the field, including the authors first describing the FNDC5/irisin browning effect, demanding loss-of-function models as relevant to understand irisin biology^28^. FNDC5 silencing definitively permitted to discern the unspecific commercial available antibody binding; and moreover, the deep analysis of FNDC5-KO cells allowed revealing its role in adipose tissue. Indeed, the most relevant finding was the discovery that lack of FNDC5 expression on C3H10T1/2 pre-adipocytes not only permitted their differentiation but also significantly increased adipogenesis. We observed that FNDC5 non-expressing cells displayed accelerated differentiation showing 2 days of advantage compared to control cells which could be followed by increased lipid accumulation and adiponectin gene expression. This advantage cannot be explained by increased proliferation rate in FNDC5-KO cells as shown. Therefore, lack of FNDC5 expression drives adipocytes fatter. This result is of relevance since it may suggest that decreased FNDC5 expression/secretion, blockage of its function through post-traslational modifications/binding inhibiting factors, or abnormal function of its receptor may participate in the development of obesity. Therefore, we postulate in one hand an “irisin-resistance” state of mature adipocytes in the occurrence of obesity impeding its beneficial effects. This resistance would be responsible of irisin over-secretion from mature adipocytes in an attempt to counterbalance the altered metabolic status. Therefore, the positive autoregulation of irisin described by us in healthy adipocyte cells and previously by others^25,29^ reinforces this hypothesis. On the other hand, we introduce a new paradigm on FNDC5/irisin regulation implicating other obese adipose secreted factors as negative regulators of FNDC5/irisin expression on differentiating pre-adipocytes enhancing adipogenesis. It was recently described that the abundance of beige adipocytes in white adipose tissue correlates positively with the response to irisin^25^; thus, theoretically, a low amount of beige adipocytes within obese white adipose tissue may explain irisin’s lack of action. Additionally, accordingly to our results, it was previously shown that FNDC5 over-expression in mice induces lipolysis and reduces the size of SAT adipocytes^30^; and more recently Zhang and collaborators have described that recombinant irisin treatment decreases adiponectin expression and adipogenesis of human white subcutaneous adipocytes^25^. It will be now necessary to discern if reduced thermogenesis and increased adipogenesis in C3H10-KO cells is an intrinsic or secretion-dependent effect. Since differentiation of C3H10-KO cells in the presence of C3H10-WT secretome, which contains intact healthy irisin, upregulates UCP-1 expression, we suggest a positive feedback of secreted irisin exerting a paracrine effect that is blunted by blocking irisin in the same WT secretomes. However, we do not know if this also applies to increased adipogenesis; therefore, this will deserve further research.

In relation to glucose metabolism, although it was shown that irisin is able to ameliorate general glucose metabolism, we did not observe any change on insulin sensitivity when performing an insulin resistance experiment (High glucose/high insulin) with FNDC5-KO adipocytes. However, certain alterations on the insulin/Akt pathway were observed in cells lacking FNDC5 expression after 10 minutes insulin stimulation such as an elevation of p-Ser473Akt, corroborating the insulin stimulation time course assayed by immunoblot in the present paper; the elevation of p-Ser380PTEN, p-Ser15P53, p-Ser386RSK2 and p-Ser112BAD, and the diminution of phospho-Thr246 proline-rich akt substrate of 40 kda (p-Thr246PRAS40).

Considering the results discussed above, it is our aim now to analyze the effect of *in vitro* FNDC5 gene silencing on human cultured adipocytes and to investigate about the presence of the excised 12 kDa irisin at intracellular level in adipocytes. In this regard, it has to be considered the first work describing FNDC5, named PeP, as a peroxisomal protein whose mRNA accumulation is induced after myoblast differentiation *in vitro* ^31^. Taking into account the increase of peroxisomes during adipogenesis, its association with adipose lipid droplets, and their ability to carry out fatty acid oxidation and lipid synthesis (ether lipids) it is reasonable to find increased FNDC5 expression during adipocyte differentiation and to find the 12 kDa excised form also at intracellular level^32^. Finally, it is indeed of interest whatsoever, to check FNDC5 silencing on muscle cells.

In conclusion, we reveal for the first time the immunoblot identification of intracellular and secreted irisin protein by knocking down FNDC5 gene in a murine model of adipocyte differentiation. Therefore, we demonstrate the unspecific binding to FNDC5/irisin of commercial antibodies, including ELISA kits, explaining the contradictory results about this subject on the bibliography and fulfilling the experts demands about this subject^28^. However, the most relevant finding comprises the decreased browning capacity and increased adipogenesis of differentiating adipocytes caused directly or indirectly by blocking adipose endogenous expression of FNDC5. Therefore, the reduction of FNDC5, PGC1α and UCP1 expression on differentiating normal adipocytes incubated with obese WAT secreted factors suggest the capacity of obese secretome to exacerbate the pathological situation. Thus, adipocyte FNDC5 intrinsic role may be of relevance during the course of pre-adipocyte differentiation impeding lipid hypertrophy.

**Figure 7:**
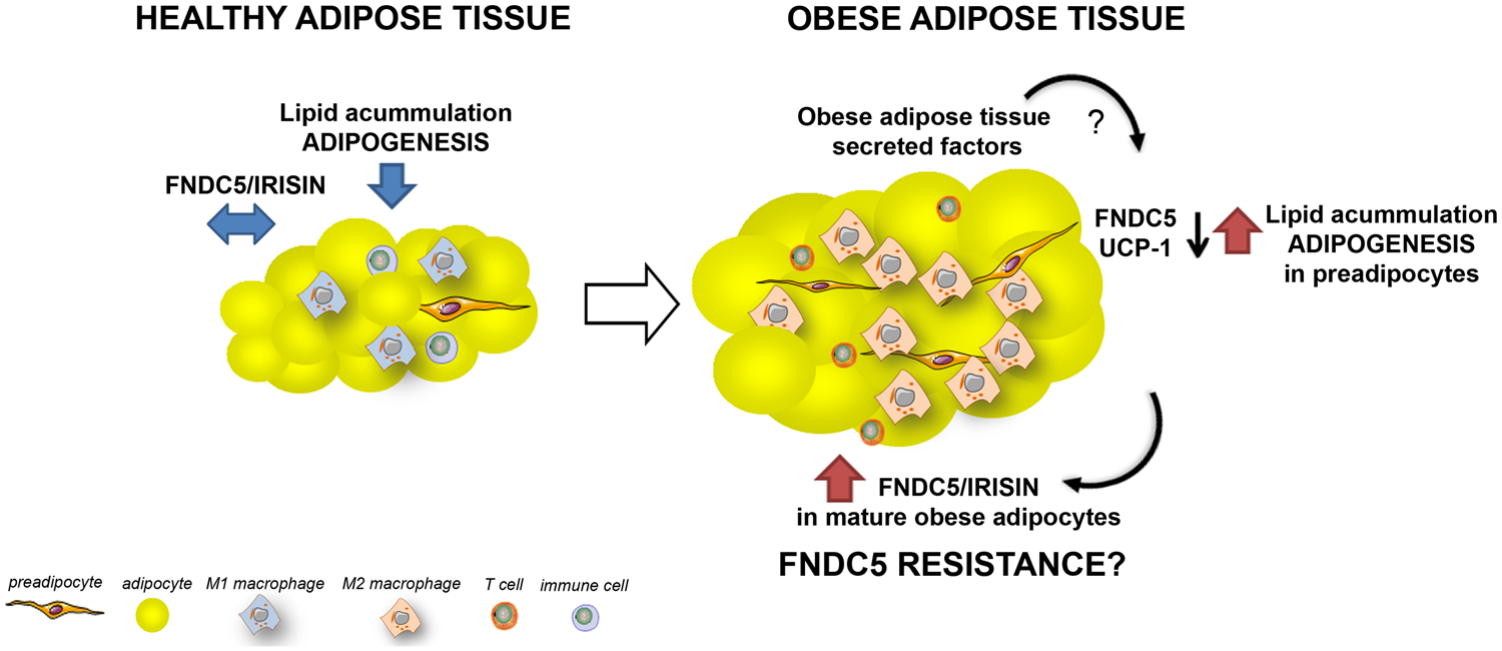
Results summary. The results and hypothesis about the role of adipose-derived FNDC5/irisin are shown in a schematic representation of healthy and obese adipose tissues. In the obese condition adipose tissue would overexpress and oversecrete FNDC5/irisin in an attempt to counterbalance the metabolic deregulation; however adipose and other peripheral tissues may suffer from FNDC5/irisin resistance. On the other hand, obese adipose tissue, and specifically obese VAT secreted factors are able to decrease FNDC5 and UCP1 expression on differentiating adipocytes promoting lipid accumulation and adipogenesis; we hypothesize that obese differentiated adipocytes are not sensitive to this inhibitory effect. Note that some cells in this representation were taken and/or modified from the Powerpoint image bank freely available at http://www.servier.com/Powerpoint-image-bank under the copy right of https://creativecommons.org/licenses/by/3.0/.

Overall, we postulate the following hypothesis (Fig. 7): in the course of obesity, secreted factors liberated by obese adipose tissue inhibit FNDC5 and UCP1 expression of differentiating pre-adipocytes promoting enhanced adipogenesis and increasing lipid accumulation. Once pre-adipocytes reach the mature state, they increase irisin expression and secretion in an attempt to counterbalance the altered metabolic status without success probably due to irisin resistance.

## Materials and Methods

### C3H10T1/2 cell culture and differentiation

The murine MSC (mesenchimal stem cell line) C3H10T1/2 cells were cultured at 37 °C under 5% CO2 in Dulbecco’s modified Eagle’s medium (Life Technologies, CA, USA) containing 10% fetal bovine serum (SIGMA-ALDRICH, MO, USA), 100 U/ml of penicillin and 100 μg/ml of streptomycin until differentiation to adipocytes as previously described^33^. In brief, after confluence differentiation was induced with induction medium (basic medium supplemented with 1 µM dexamethasone, 0.5 mM isobutylmethylxanthine, 1 µM rosiglitazone and 5 µg/mL insulin). Two days after induction, the medium was changed to basic medium with insulin. Accumulation of cytoplasmatic triglyceride in these cells was detected by staining with Oil Red O (SIGMA-ALDRICH, MO, USA).

### FNDC5 silencing of C3H10T1/2 cells

pLKO.1 lentiviral expression plasmids containing short hairpin RNAs against FNDC5 was purchased from Sigma (Mission shRNA, SIGMA-ALDRICH, MI, USA). Stable FNDC5 knockdown C3H10T1/2 cell lines were generated by transducing lentiviral particles carrying various target FNDC5 shRNAs. The viral particles were packaged by transfecting FNDC5 shRNAs together with lentiviral packaging mix (SIGMA-ALDRICH, MO, USA) in HEK 293 cells according to the manufacturer’s instructions. The target sequences for hairpins directed against human FNDC5 (NM_027402) were: TRCN0000098525:CCGGCCCTCTGTGAACATCATCAAACTCGAGTTTGATGATGTTCACAGAGGGTTTTTG;TRCN0000098526:CCGGCGAGCCCAATAACAACAAGGACTCGAGTCCTTGTTGTTATTGGGCTCGTTTTTG;TRCN0000098527:CCGGGACACAGAATATATCGTCCATCTCGAGATGGACGATATATTCTGTGTCTTTTTG;TRCN0000098528:CCGGGCTCTCTTCTGCCGCCAGTATCTCGAGATACTGGCGGCAGAAGAGAGCTTTTTG;TRCN0000098529:CCGGGTGCGGATGCTCCGGTTCATTCTCGAGAATGAACCGGAGCATCCGCACTTTTTG. GFP shRNAs (SIGMA-ALDRICH, MO, USA) was used as control to prove the correct viral packaged particles introduction into the cells.

### Human serum and adipose tissue acquisition

The human blood and adipose tissue specimens were obtained with written informed consent approved for this particular study (Ref.2013/425) by the Comité Ético de Investigación Clínica de Galicia - CEIC de Galicia, (Spain) according to the Declaration of Helsinki. Adipose tissue was obtained from healthy normal weight volunteers (body mass index <35) who underwent cholecystectomy surgery and from obese patients (body mass index >35) who underwent laparoscopic gastrectomy or bypass surgery. The visceral fat was located in the hypogastric region around the internal organs, and the subcutaneous fat was located in the mesogastric region. The tissues were transported from the operating room to the laboratory in sterile PBS buffer with penicillin (100 U/ml) and streptomycin (100 μg/ml). Secretome and tissues were collected and processed for immunodetection and functional studies as previously described^17,34^. Briefly, tissues were processed to eliminate any contaminant and washed thoroughly in PBS under sterile conditions in a flow laminar hood. Next, fat pieces were centrifuged in a 25 ml tube with 20 ml of PBS for 5 min at room temperature to remove blood cells and cell debris. Fat pieces of 2 g were incubated in 6 well cell culture dishes (Iwaki, Tokyo, Japan) in 4 ml of serum free medium that was changed twice every two hours and again after 16 h. Fresh serum/phenol red free DMEM medium was added and incubated for 24 h to obtain final secretomes.

Human SVF and mature adipocytes isolation was performed by digesting adipose tissue (1 g minced in little pieces) with 0.2% collagenase type I (Sigma-Aldrich; St Louis, MO). The digested sample was centrifuged at 400 g during 5 minutes to separate floating mature adipocytes from SVF that are compacted at the bottom of the tube.

### RNA isolation and quantitative real-time PCR

Total RNA was isolated from C3H10T1/2 cells using GeneJET RNA Purification kit (Thermo Scientific, MA, USA) according to the manufacturer’s recommendations. Quantitative real-time PCR was performed using a Real Time PCR Systems Step One Plus (Applied Biosystems/Thermo Fisher Scientific, MA, USA) with specific Taqman qRT-PCR primers as shown in Table 1. The levels of gene expression were normalized using β-actin in C3H10T1/2 cells as housekeeping gene, and were expressed with respect to the average value in the control group.

**Table 1:** Taqman qRT-primers commercial reference for each analysed gene.

GENE	MOUSE PRIMERS
β-actin	Mm00607939_s1
FNDC5	Mm01151543_m1
ADIPOQ	Mm00456425_m1
PPARγ	Mm00440940_m1
PGC1α	Mm01208835_m1
UCP1	Mm01244861_m1
SIc2a4 (GLUT4)	Mm00436615_m1

### Immunochemistry

Differentiated and non-differentiated C3H10T1/2 cells were cultured in glass slides inside sterile Petri dishes. Cells were fixed in 96% ethanol during 30 minutes before epitope retrieval in EnVision FLEX target solution (pH 9) for 20 min in a microwave oven. Then, the slides were cooled to room temperature for 10 minutes and submerged in Dako wash buffer for 5 min. The immunostaining protocol included the following steps: (1) Incubation in EnVision FLEX peroxidase-blocking reagent (Dako) for 10 min; (2) incubation with anti- FNDC5 rabbit monoclonal (RabMab Abcam (Cambridge, UK) at a dilution of 1/100 in EnVision FLEX Antibody Diluent overnight at 36 °C; (3) secondary goat anti-rabbit IgG-HRP (Santa Cruz Biotechnology) during 30 minutes; (4) incubation in substrate working solution (mix) (3,3′-diaminobenzidine tetrahydrochloridechromogen solution) (Dako) for 10 min; (5) EnVision FLEX hematoxylin (Dako) for 9 min. For immunofluorescence a secondary antibody Alexa Fluor® 488 in 1% BSA/PBST was used (1 h, RT), and DAPI to counterstain the cell nuclei (Invitrogen). Digital images of cells were acquired with a Zeiss Axio Vert.A1 fluorescence microscope (Carl Zeiss AG, Oberkochen, Germany).

For human adipose samples tissues were fixed in 10% formalin and analyzed using the same protocol as described for cell line but using a linker to improve the signal due to the high lipid fraction contained in human adipocytes.

### Immunoblotting

Protein extracts from whole tissue samples and secretomes were processed as previously described^17,34^. For 1-D western blotting, 50 μg of adipose tissue/C3H10T1/2 cells secreted proteins, and 30 μg of whole tissue/cell extracts from at least four independent experiments were separated in SDS-PAGE gels and electroblotted onto PVDF membranes. Equal loading was confirmed by membrane staining with Ponceau S (Sigma-Aldrich; St Louis, MO) in the case of secretome protein extracts, or by measuring the amount of GAPDH in whole tissue protein extracts. For 2-D western blotting, 50 μg of protein were taken into a final volume of 120 μl in 2-DE sample buffer containing 5 M urea, 2 M thiourea, 2 mM tributylphosphine, 65 mM DTT, 65 mM CHAPS and 0.15 M NDSB-256. Ampholytes were added to the sample at 0.1% servalyte 3–10, 0.05% servalyte 2–4 and 9–11 (SERVA, Heidelberg, Germany). 3–10 NL 7 cm IPG strips (BioRad, CA) were actively rehydrated in the sample and IEF was carried out in a Protean IEF cell following the manufacturer protocol (BioRad, CA). Following focusing, the IPG strips were immediately equilibrated for 20 min in 4 M urea, 2 mM thiourea, 12 mM DTT, 50 mM Tris pH 6.8, 2% SDS, and 30% glycerol. The IPG strips were placed on top of the second dimension gels (15% SDS-PAGE) and embedded with 1% melted agarose. Electrophoresis and immunodetection was conducted as for 1-D western blot.

Primary anti- FNDC5 Rabbit monoclonal (ab174833, aa 50–150) and anti-UCP-1 (ab10983) were purchased from Abcam (Cambridge, UK); anti-PPARγ, anti-Akt and anti-p-Akt (Ser473) from Cell Signaling Technology (MA, USA); anti-PGC1α, FABP3 and adiponectin from Santa Cruz Biotechnology Inc. (CA, USA); and anti-GAPDH was purchased from Life Technologies Ltd (Paisley, UK).

To asses FNDC5 antibody specificity, the immunizing/blocking peptide (FNDC5 peptide ab204133) was purchased from the same manufacturer as the antibody (Abcam, Cambridge, UK). Antibody blocking was performed by adding 2 ug of peptide for 30 minutes at room temperature.

For FNDC5 immunoprecipitation, 800 ug of total protein was incubated with 2 µg of anti-FNDC5 monoclonal antibody (Abcam, Cambridge, UK) overnight at 4 °C, followed by the addition of 60 µL of 50% protein A/G-agarose beads (Santa Cruz Biotechnology) for 2 h at 4 °C. After incubation, beads were washed three times with RIPA buffer. The pelleted beads were resuspended in Laemmli sample buffer and boiled at 95 °C for immunoblotting.

To perform the AKT pathway phosphorylation array for semi-quantitative detection of 18 phosphorylated proteins cells were deprived 2 hours in low glucose (1 g/L) cell culture medium followed by 10 minutes stimulation with 100 nM insulin following the manufacturer instructions (RayBio Biotech, Inc GA, USA).

### Measurement of FNDC5/irisin by ELISA

The quantitative measurement of irisin in blood plasma, cell lysates and secretomes was performed using a commercial competitive enzyme-linked immunosorbent assay (ELISA) AG-45A-0046YEK-KI01 (Adipogen, Switzerland) with a sensitivity of 1ng/ml and a detection range of 0.001–5 µg/mL according to manufacturer’s instructions. The absorbance from each sample was measured in duplicate using a spectrophotometric microplate reader at wavelength of 450 nm (Versamax Microplate Reader; Associates of Cape Cod Incorporated, East Falmouth, MA).

### ExCelligence proliferation and differentiation analysis

Cell proliferation and differentiation analysis was assessed by using the label-free and real-time monitoring xCELLigence system (ACEA Biosciences, San Diego, CA). Under this platform, Cell index (CI) was the parameter used to represent cell status based on the measured electrical impedance^35^ Briefly, 3000 cells/well were seeded (E-plates 16 wells) and incubated for six days to observe differences in the proliferation pattern. In the case of the differentiation analysis, cells were allowed to reach a confluence of about 90% before adding the differentiation cocktail as described above.

### Functional studies with conditioned secretomes

Cells were seeded to confluence and differentiated with the standard adipogenic cocktail for two days. After this induction, cells were treated with medium containing 10% serum-free conditioned secretome for 24 hours with or without previous blockage of FNDC5/irisin. FNDC5/irisin blocking was performed by incubating obese secretomes with 2 µg of FNDC5 antibody (Anti-FNDC5 rabbit monoclonal Abcam) during 30 minutes at room temperature. Conditioned secretome treatments were performed in three experimental settings: (a) control and FNDC5-KO C3H10T1/2 cells with human VAT and SAT obese secretome; (b) control and FNDC5-KO C3H10T1/2 cells with human VAT and SAT healthy secretome; and (c) FNDC5-KO C3H10T1/2 cells with C3H10T1/2 control cell secretomes. Treatments with plasma were performed by using the plasma from the corresponding secretome patient at 10% in serum-free medium following the same protocol as for secretomes. Functional experiments of Fig. 4 performed on totally differentiated cells (day 10) were done as the above by treating cells with 10% obese VAT and SAT secretomes for 24 hours.

### Insulin resistance model

High glucose and high insulin (HGHI) model was performed as previously described^36^. Briefly, cells were differentiated until day 10, washed three times with PBS and incubated during 2 hours in low glucose (1 g/L) cell culture medium. Cells were then cultured in high glucose (4.5 g/L) and high insulin (100 nM) serum-free medium for 24 hours. After three PBS washing steps, cells were stimulated with insulin at 100 nM for 10 minutes.

### Statistical analysis

Results are presented as mean ± SE of at least three independent experiments, and each experiment was conducted at least in triplicate. Statistical significance among multiple groups was analyzed by one-way Anova-Kruskall Wallis test followed by Dunn’s multiple comparison test or two-way ANOVA with post hoc Bonferroni correction for multiple comparisons, and Mann-Whitney U test for comparison of results between two groups using GraphPad Prism 5 software. P ≤ 0.05 was considered statistical significant.

## Electronic supplementary material

Supplementary info
