# Supplementary material for: Lack of Adipocyte-Fndc5/Irisin Expression and Secretion Reduces Thermogenesis and Enhances Adipogenesis
Source: Sci Rep. 2017 Nov 24;7:16289. doi: 10.1038/s41598-017-16602-z (PMC5701255; doi:10.1038/s41598-017-16602-z)
Supplement: Supplementary file 1 — Supplementary info [file 41598_2017_16602_MOESM1_ESM.docx]

***LACK OF ADIPOCYTE-FNDC5/IRISIN EXPRESSION AND SECRETION REDUCES THERMOGENESIS AND ENHANCES ADIPOGENESIS***

Perez-Sotelo D ^1^, Roca-Rivada A^1^, Baamonde I^2^, Baltar J^2^, Castro AI^3,6^, Domínguez E^4^, Collado M^5^, Casanueva FF^3,6^, and Pardo M*^1, 3^.

1. Grupo Obesidómica, Área de Endocrinología, Instituto de Investigación Sanitaria de Santiago de Compostela (IDIS), Xerencia de Xestión Integrada de Santiago (XXIS/SERGAS), Santiago de Compostela, Spain

2. Servicio de Cirugía General, Xerencia de Xestión Integrada de Santiago (XXIS/SERGAS), Santiago de Compostela, Spain.

3. CIBER Fisiopatología Obesidad y Nutrición, Instituto de Salud Carlos III, Spain Laboratorio de

4. Grupo Biofarma C026, Centro de Investigación en Medicina Molecular e Enfermidades Crónicas (IDIS-CIMUS), Universidade de Santiago de Compostela.

5. Grupo Células Madre en Cáncer y Envejecimiento, Área de Oncología, Instituto de Investigación Sanitaria de Santiago de Compostela (IDIS), Xerencia de Xestión Integrada de Santiago (XXIS/SERGAS), Santiago de Compostela, Spain.

6. Endocrinología Molecular y Celular, Instituto de Investigación Sanitaria de Santiago (IDIS), Xerencia de Xestión Integrada de Santiago (XXIS/SERGAS), Spain

* To whom correspondence should be addressed: maruxapardo@hotmail.com

SUPPLEMENTARY FIGURE 1


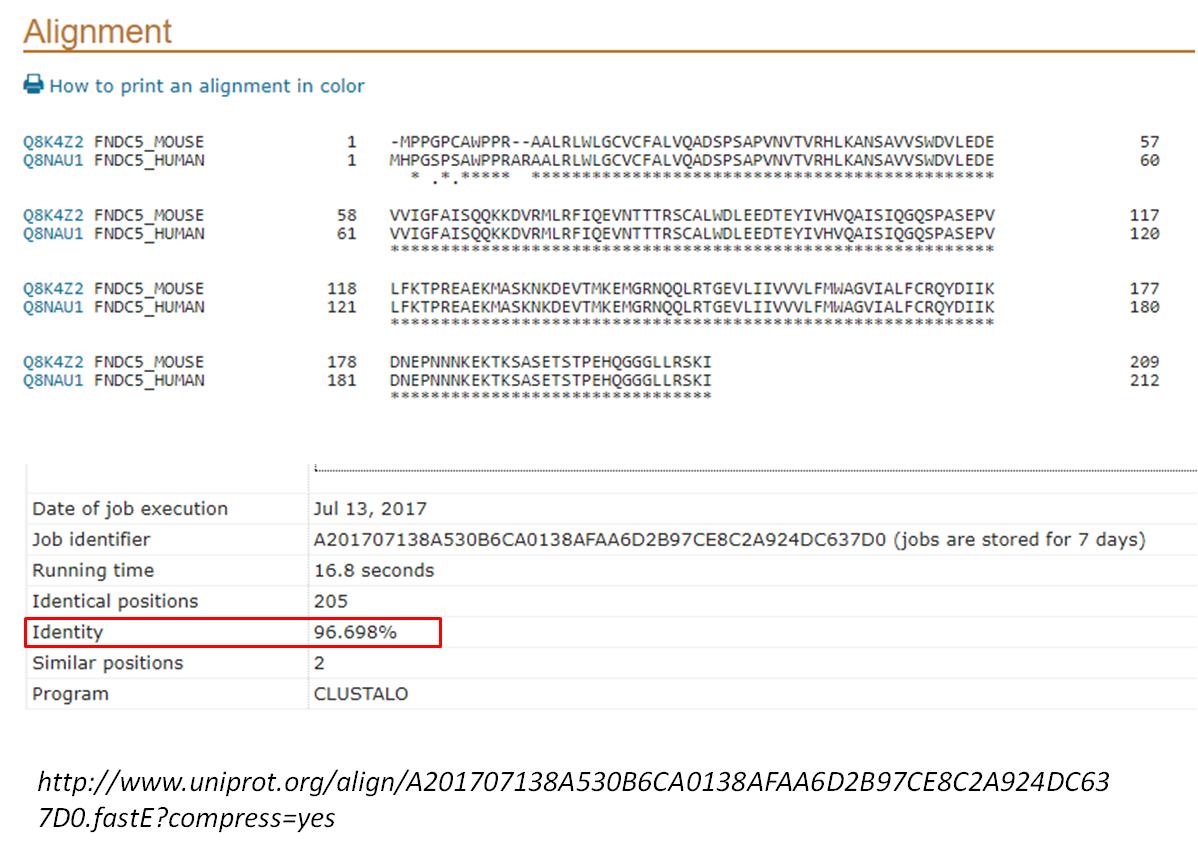


SUPPLEMENTARY FIGURE 2


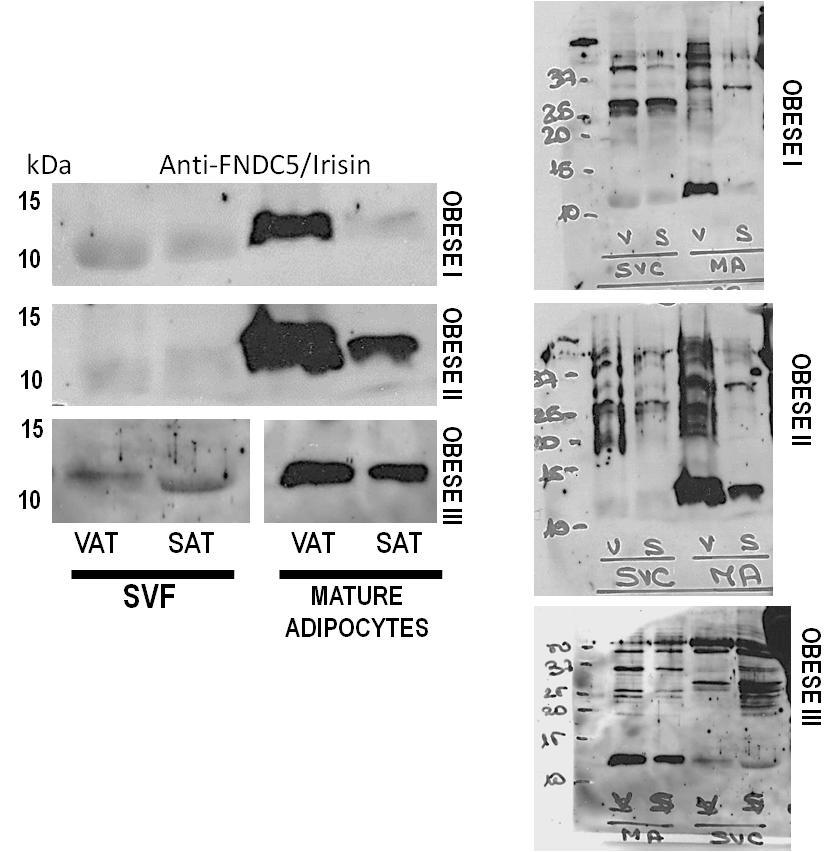


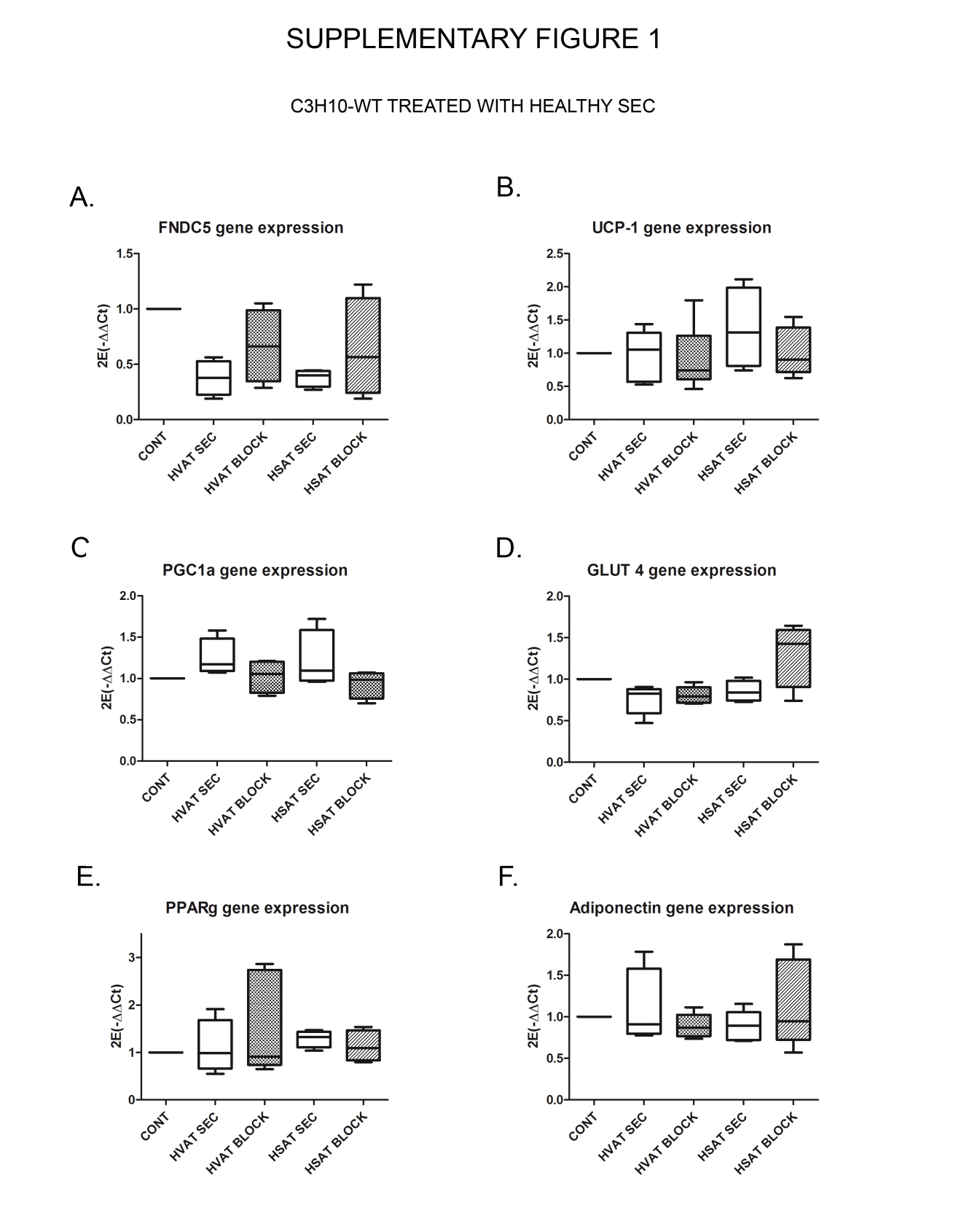


SUPPLEMENTARY FIGURE 3


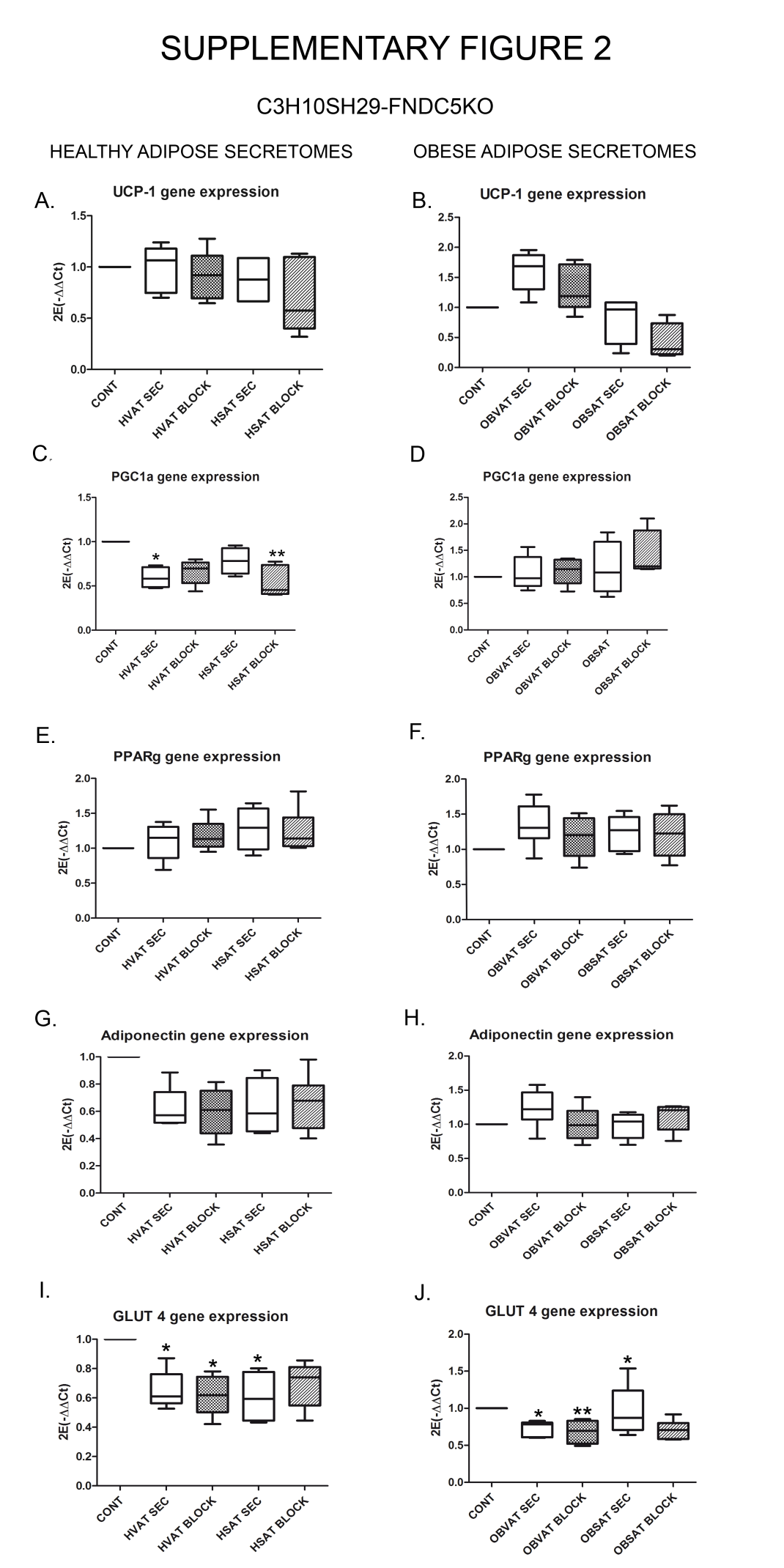


SUPPLEMENTARY FIGURE 4

SUPPLEMENTARY FIGURE 5

UCP-1

Adiponectin

PPARg

SUPPLEMENTARY FIGURE 3


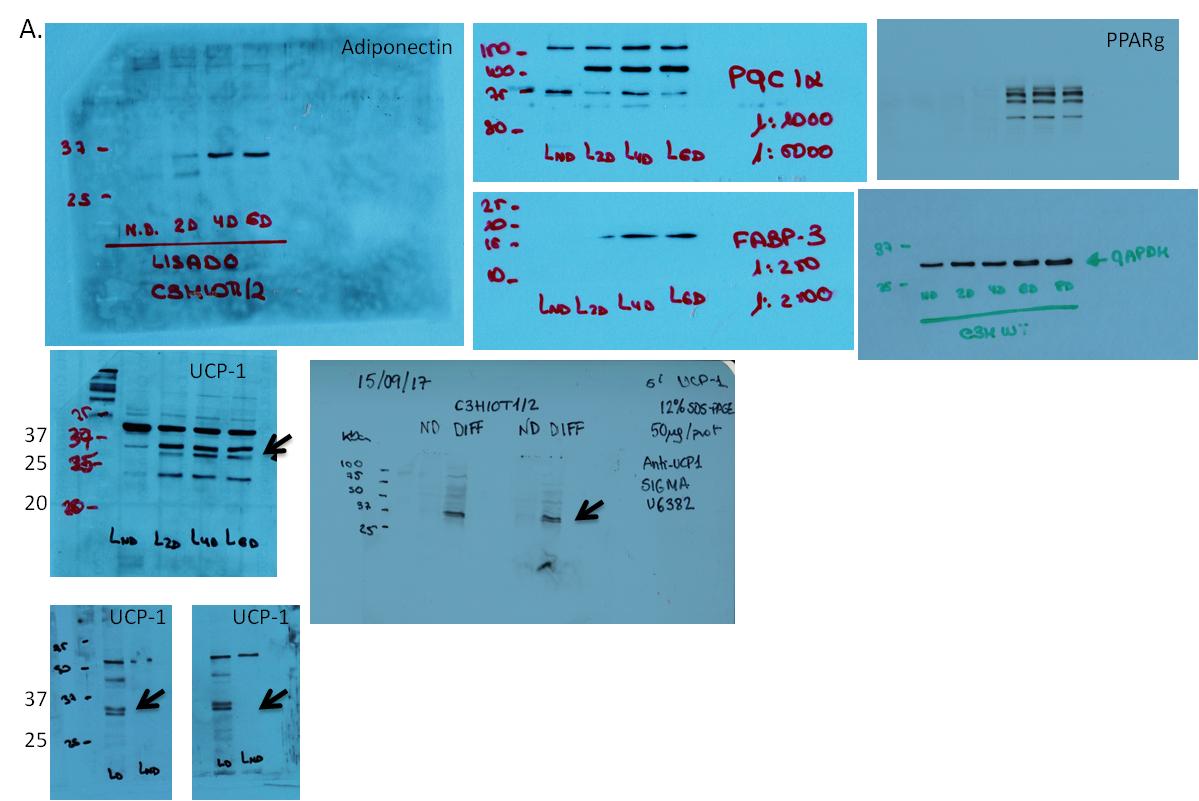


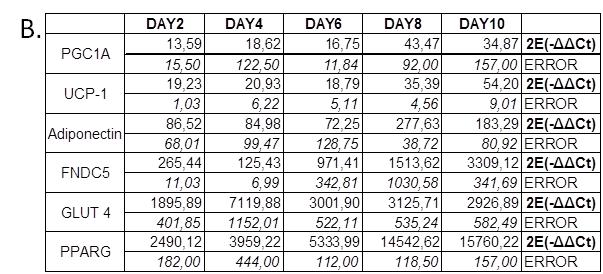


SUPPLEMENTARY FIGURE 6

A

B

SUPPLEMENTARY FIGURE 7


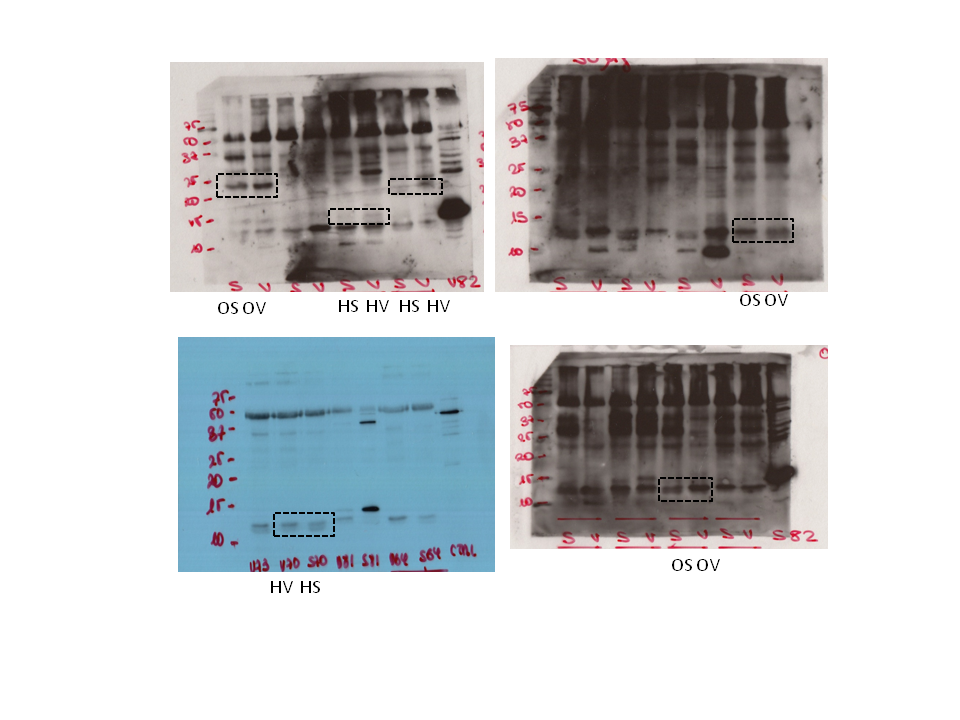


SUPPLEMENTARY FIGURE 8

FNDC5-KO

A

CONTROL

Control

B

C


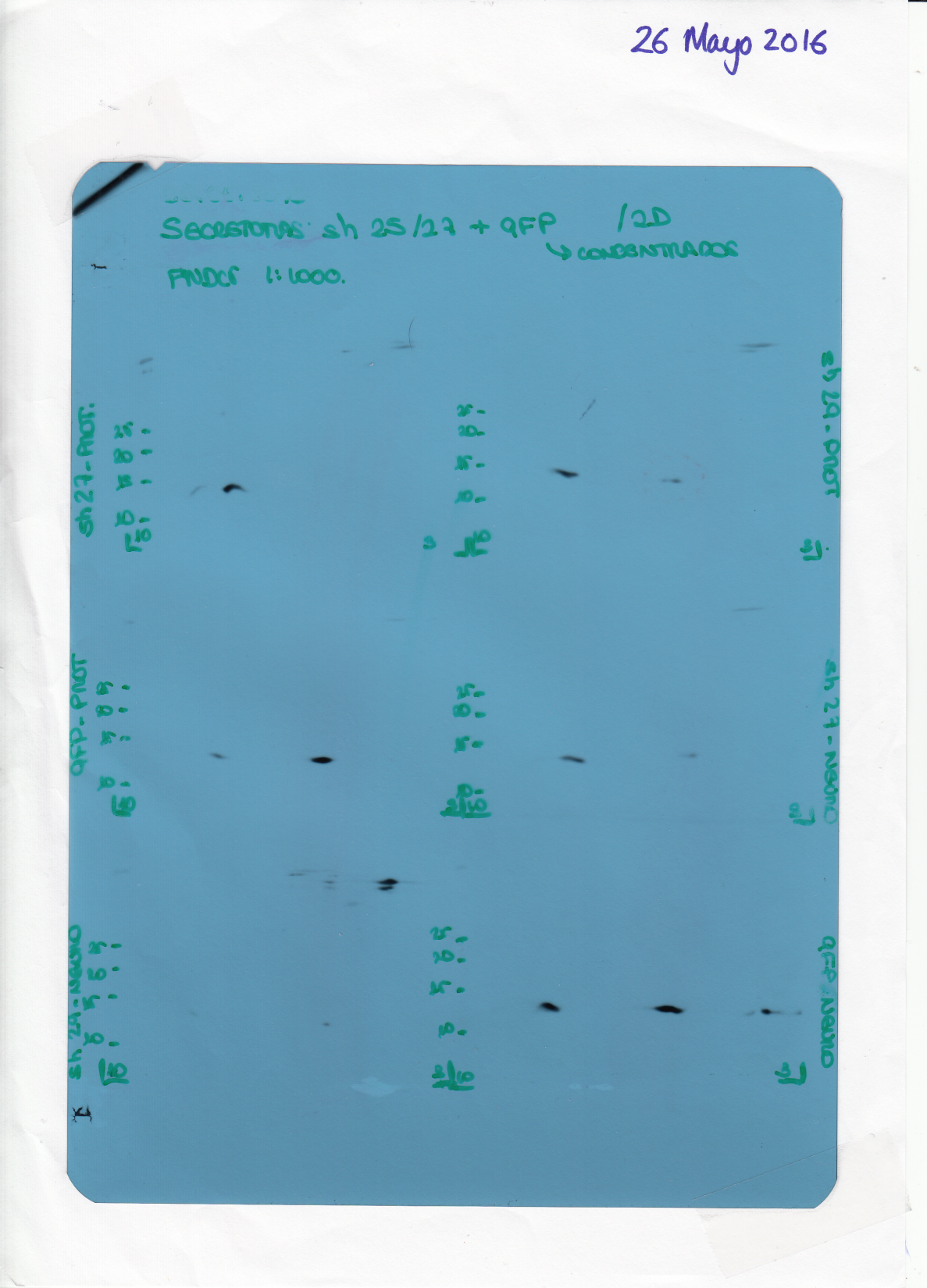


FNDC5-KO

D


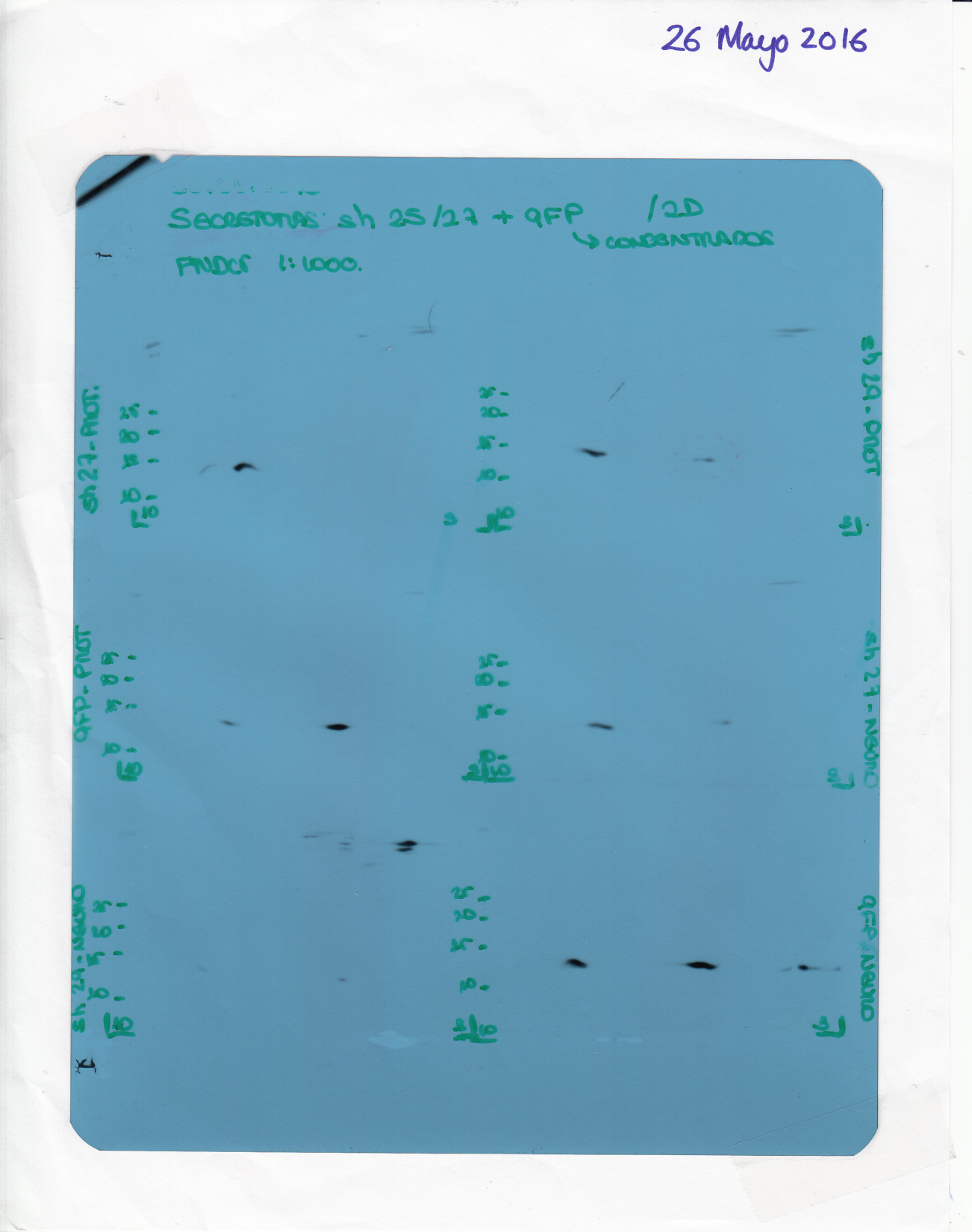


SUPPLEMENTARY TABLE 1

**Supplementary Figure 1.** Mouse and human FNDC5 are 97% identical. Alignment of mouse and human FNDC5 protein sequences blasted at Uniprot.

**Supplementary Figure 2.** Only human mature adipocytes secrete irisin. Irisin 12 KDa band is detected mainly in the secretome from mature adipocytes; only a faint band is shown in the secretome of the stromal vascular fraction (SVF) of human obese adipose tissues. Irisin detection on the secretome of SVF and mature adipocytes fractions from 3 VAT and SAT samples of 3 independent obese individuals is shown (cropped and full length images).

**Supplementary Figure 3.** Human healthy VAT and SAT secreted factors do not affect normal adipose cells gene expression during differentiation. **A:** FNDC5; **B:** UCP1; **C:** PGC1α; **D:** GLUT4; **E:** PPARγ; and **F:** Adiponectin gene expression in murine C3H10T1/2 pre-adipocytes differentiated in the presence of healthy VAT and SAT secreted factors with or without blocking soluble FNDC5/irisin (*n=*4 independent human obese secretomes). Histograms show the quantitative expression levels towards control non-treated cells for data normalization [One way ANOVA Kruskal-Wallis test followed by Dunn´s multiple comparison].

**Supplementary Figure 4.** FNDC5-KO adipose cells gene expression is not affected by human healthy and obese VAT and SAT secreted factors. **A-B:** UCP1; **C-D:** PGC1α; **E-F:** PPARγ, **G-H:** Adiponectin and **I-J:** GLUT4 gene expression is shown in FNDC5-KO C3H10T1/2 pre-adipocytes differentiated in the presence of healthy and obese VAT and SAT secreted factors with or without blocking soluble FNDC5/irisin (*n=*4 independent human secretomes). Histograms show the quantitative expression levels towards control non-treated cells for data normalization [One way ANOVA Kruskal-Wallis test followed by Dunn´s multiple comparison]. * p<0.05, **P<0.01, and *** p<0.001 versus control non-treated cells.

**Supplementary Figure 5.** A. Full-legth Western blot films showing representative expression of Adiponectin, PGC1a, FABP-3, PPARg, UCP-1 (including immunodetection with a different antibody-SIGMA U6382) and GAPDH during C3H10T1/2 differentiation into mature adipocytes in manuscript Figure 1; B. Table showing 2E(-∆∆Ct) values and standard errors for each analyzed gen in Figure 1.

**Supplementary Figure 6.** Full-length 2-DE Western blot films showing FNDC5 immunoprecipitation and detection in differentiated and non-differentiated C3H10T1/2 cells in Figure 1F (A); and FNDC5 detection in cell lysates with and without blocking the detection antibody in Figure 1G (B).

**Supplementary Figure 7.** Full-length Western blot films showing FNDC5 isoforms in human obese adipose tissue secretomes. Representative bands shown in Figure 2I are shown in boxes.

**Supplementary Figure 8.** Full-length 2-DE Western blot films of FNDC5 in C3H10T1/2 control cells and silenced for FNDC5 expression. Cell lysates are shown in A and B and in the manuscript in Figure 5C; and secretomes in C and D, Figure 5D in the manuscript.

**Supplementary Table 1.** Anthropometric parameters of the study subjects.
